# The effect of consuming nuts on cognitive function: a systematic review and meta-analysis of randomized clinical trials

**DOI:** 10.3389/fnut.2024.1463801

**Published:** 2024-09-04

**Authors:** Mahdi Moabedi, Mohammadreza Aliakbari, Shima Erfanian, Aliyu Tijani Jibril, Alireza Milajerdi

**Affiliations:** ^1^Student Research Committee, Kashan University of Medical Sciences, Kashan, Iran; ^2^Student Research Committee, Isfahan University of Medical Sciences, Isfahan, Iran; ^3^Department of Community Nutrition, School of Nutritional Sciences and Dietetics, Tehran University of Medical Sciences, Tehran, Iran; ^4^Research Center for Biochemistry and Nutrition in Metabolic Diseases, Institute for Basic Sciences, Kashan University of Medical Sciences, Kashan, Iran

**Keywords:** cognition, adults, meta-analysis, clinical trials, nuts

## Abstract

**Background:**

Results from clinical trials investigating the effect of nuts consumption on cognition are conflicting. We decided to conduct the current meta-analysis to summarize all available evidence on the effect of consuming nuts on cognition scores.

**Methods:**

We conducted a comprehensive search in the online databases using relevant keywords up to June 2024. We included all the published Randomized clinical trials (RCTs) investigating the effect of nuts, compared to control, on cognition scores.

**Results:**

Overall, 5 trials were included with a total sample size of 928 adults. Based on 6 effect sizes from these 5 trials, we did not find a significant effect of nuts on cognition function [Standardized Mean Difference (SMD): 0.27, 95% CI: −0.65 to 1.19, *p* = 0.57].

**Conclusion:**

Our review could not find a significant effect of nuts on cognition function. Future high-quality RCTs with larger sample sizes should be conducted to shed light on the impact of nuts on cognition.

## Introduction

Cognitive function is a term that refers to mental processes involved in giving reasons, the inception of knowledge, and the processing of information. Cognitive functions include the domains of perception, language abilities, learning, attention, decision-making, and memory (1). It means a set of mental functions that lead to perceiving and reacting, processing and understanding, making decisions, and producing appropriate responses to the environment. The quality of human life is significantly impacted by cognitive performance from two perspectives. 1. Loss or reduction of this ability leads to Alzheimer’s. 2. In various mental illnesses, such as major depressive disorder, schizophrenia, and bipolar disorder, cognitive function is impaired. So, if we can improve cognitive function, we can also enhance mental health and address Alzheimer’s disease (2). Cognitive impairments such as Alzheimer’s and dementia are major concerns (3). The prevalence of Alzheimer’s rises with age: 5.3% of elderlies aged from 65 to 74 years, 13.8% of elderlies aged from 75 to 84 years, and 34.6% of elderlies aged 85 or older have Alzheimer’s disease (4).

Nuts are foods with high amounts of nutrients such as antioxidant and anti-inflammatory compounds. Nuts contain biologically active compounds and nutrients, such as unsaturated fatty acids, high-quality protein, a diversity of vitamins and critical minerals, phenolic compounds, phytosterols, and fiber which may have beneficial effects on brain functions (5). Several RCTs show a significant effect of nuts on the improvement of cognition performance. Barbour et al. study in 2016 suggests that High-oleic peanut supplementation may regulate some functions in the brain as it may improve cognitive function in people with cognitive impairment (6). On the other hand, Mustra Rakic et al. found no significant difference in change in cognition function scores following consumption of almonds among the three groups included in the study (7). In a systematic review by Theodore et al., it was concluded that the benefit of nut intake on cognition may be more significant in people with a higher risk of cognitive impairment (8). However, no meta-analysis was conducted on the included studies in this systematic review.

Therefore, regarding that there are multiple randomized clinical trials and by the time of the current review there is no meta-analysis summarizing the effect of nuts consumption on cognitive performance, we decided to conduct this meta-analysis.

## Method

This study was performed based on the PRISMA protocol for systematic reviews and meta-analyses (Supplementary Table S1). PROSPERO registration ID: CRD42023455924.

### Search strategy

We conducted a systematic search in the online databases of Web of Science, Scopus, PubMed, and Google Scholar up to June 2024, with the following keywords: [(nut OR almond OR cashew OR “tree nut” OR peanut OR pecan OR “pine nut” OR pistachio OR macadamia OR “peanut butter” OR hazelnut OR walnut) AND (cognition OR executive “executive function” OR “cognitive control” OR intelligence OR memory OR attention OR metacognition OR cognit* OR neuropsych* OR psychomotor OR learning OR language OR “executive function” OR attention OR “social cognition”)]. The complete search strategy is presented in Supplementary Table S2.

We made no restriction on the time or language of publications. In addition, the reference list of the relevant papers was reviewed to avoid missing any publication. Duplicate papers were also removed.

### Inclusion criteria

Studies that met the following criteria were included: (1) randomized controlled clinical trials, (2) done on adults, (3) trials that intervened with nuts in different types such as peanut, almond, soy nut, walnut, Brazil nut, and their products, (4) clinical trials with at least 12 weeks of intervention duration, and (5) trials that reported mean changes with their standard deviations (SDs) of cognition function scores throughout the trial for both intervention and control groups or required information for calculation of those effect sizes. If more than one article was published for one dataset, we included only the most complete one.

### Exclusion criteria

We excluded observational studies, review articles and ecological studies. Clinical trials without a control group were also excluded. Non-randomized trials were also excluded. Moreover, unpublished studies and grey literature were removed during the screening process.

### Data extraction

Two independent investigators conducted screening and data extraction, looking for the following information from included studies: first author’s name, publication year, individuals’ characteristics (mean age and sex), sample size (control and intervention groups), study design, type of nuts which was consumed, the amount of nut, intervention duration, and mean changes and their SDs or alternative convertible data of cognition function scores throughout the trial for the intervention and control groups.

### Quality assessment

The risk of bias for each included study was examined by the Cochrane quality assessment tool (9). This tool contained seven domains including allocation concealment, reporting bias, random sequence generation, performance bias, detection bias, attrition bias, and other sources of bias. Each domain was given a “high risk” score if the study comprised methodological defects, a “low risk” score if it had no defect for that domain, and an “unclear risk” score if there was no sufficient data to determine the risk. The overall risk of bias for each RCT was considered: (1) Low; if all domains were “low risk,” (2) Moderate; if one or more domains were “unclear risk,” and (3) High; if only one or more than one domain was “high risk.” Quality assessment is presented in Supplementary Table S3.

### Statistical analysis

We used mean changes and SDs of depression scores in the nuts and control groups to calculate Standardized Mean Differences (SMD). When mean changes were not reported, they were calculated from final and baseline reports of that variable. We also converted standard errors (SEs), 95% confidence intervals (CIs), and interquartile ranges (IQRs) to SDs using the suitable formula by the method introduced by Hozo et al. (10). We used a random-effects model, that takes between-study variations into account, for final analyses. Between-study heterogeneity was determined by the *I*^2^ statistic and Cochrane’s *Q* test. *I*^2^ values >50% were considered significant between-study heterogeneity.

To find probable sources of heterogeneity, we performed different subgroup analyses by the predefined variables including type of nut (tree nuts vs. non-tree nuts), mean age of participants (<60 years vs. >60 years), study location (Europe vs. non-Europe), and duration of the intervention (>6 months vs. ≤6 months). We used sensitivity analysis to detect each study on the overall effect sizes. Eggar’s regression test examined the possibility of publication bias. We carried out the current meta-analysis using Stata software, version 17 (Stata Corp). We considered a *p*-value less than 0.05 statistically significant.

## Results

Excluded studies: From 4,360 records that were found in our primary search, there were 991 duplicate articles that we excluded. After screening the remaining 3,234 publications based on title and abstract, 3,222 unrelated articles were also removed. Then, 12 publications remained for more evaluation of the full text. Out of those 12 studies, two papers were excluded due to the simultaneous Mediterranean diet (11, 12), one article was excluded since no interventions of nuts were conducted (13), and three articles were removed due to not reporting some of the required data (14–16). Finally, we excluded two papers since tests were not directly examining cognitive function but factors related to it such as stress and memory (17, 18).

**Figure 1:**
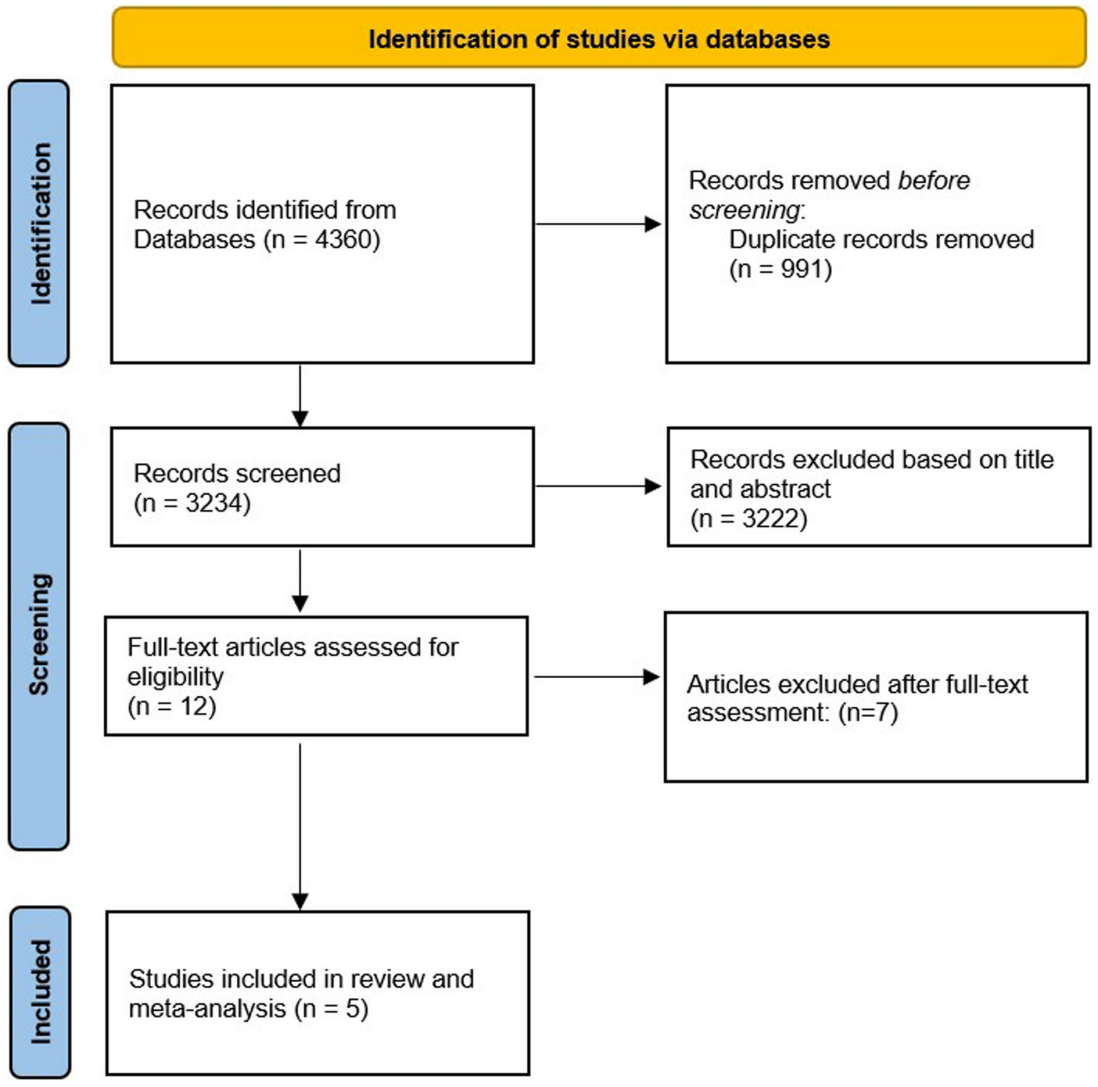
The flow diagram of the study screening and selection.

After these exclusions, five eligible RCTs remained to be included in the current systematic review and meta-analysis (6, 7, 19–21). The PRISMA flow diagram of study selection is illustrated in Figure 1.

Study characteristics: The characteristics of the five clinical trials that were included in the current systematic review and meta-analysis are outlined in Table 1. These papers were published between 2015 and 2021 and all the studies were conducted on both sexes. The sample sizes of studies varied from 20 to 657 individuals, which resulted in a total sample size of 928 individuals. The mean age of participants was between 31 ± 13 and 77 ± 6 years. The duration of intervention ranged from 12 weeks to 2 years across included trials.

**Table 1:** Summary of clinical trials on the effects of nuts on cognition status in adults.

Author, year	Design	Sex	Participants, *n*	Age, year^a^	Intervention		Daily amount of administered nut	Duration	Cognition test	Outcomes (score changes)	
					Intervention group	Control group				Treatment group	Control group
Cardoso et al. 2016 (22)	RA/SB/parallel	M/F	Int: 11, Con: 9	77 ± 6	Brazilian nuts	No placebo	100 g	16 months	CERAD	1.1 ± 8.63	−3.1 ± 4.28
Barbour et al. 2016 (6)	RA/DB/crossover	M/F	Int: 32, Con: 29	65 ± 7	Peanuts	No placebo	84 g in M	12 weeks	RAVLT target	4.3 ± 2.15	3.6 ± 1.96
							56 g in F				
							(15% of E)				
Dhillon et al. 2017 (19)	RA/DB/parallel	M/F	Int: 43, Con: 43	31 ± 13	Almond	Placebo (Carb)	53 g	12 weeks	CPT	15.97 ± 3.27	7.93 ± 3.97
Rakic et al. 2021 (7)	RA/SB/parallel	M/F	Int (1.5 oz. nut): 19	62 ± 6	Almond	Placebo (3.5 oz. (99.22 g) matched snacks)	1.5 oz. (42.5 g)	6 months	FAMS	1.5 oz. almond 0.8 ± 1.49 3 oz. almond 1.9 ± 0.96	2.4 ± 0.91
			Int (3 oz. nut): 24, Con: 17				3 oz. (85 g)				
Sala-Vila et al. 2020 (21)	RA/SB/parallel	M/F	Int: 336, Con: 321	69 ± 0.2	Walnut	Placebo (matched meal)	15% of E input	2 years	Global cognition	−0.072 ± 0.12	−0.076 ± 0.11

RA, randomized; DB, double-blinded; SB, single-blinded; Int, intervention; Con, control; M, male; F, female; MI, mild; SRP, skin roasted peanuts; PB, peanut butter; CERAD, Consortium to Establish a Registry for Alzheimer’s Disease; RAVLT, Rey Auditory Verbal Learning Test; CPT, Cognition performance test; g, grams; E, energy; Carb, carbohydrate.

a Values are mean ± SD years.

The included studies used different assessment tools for reporting changes in cognitive function. One study used the consortium to establish a registry for Alzheimer’s disease (CERAD) (22). This test assesses the characteristics which they first get impaired following Alzheimer’s occurrence (e.g., naming and verbal memory) and it has been translated into many languages and is widely being used for cognition assessment with an acceptable sensitivity (23, 24). Rey auditory verbal learning test (RAVLT) is another tool that consists of 9 trials that examine recognition, and immediate and delayed memory as factors for cognitive function which Barbour et al. have assessed cognition by in their trial (6, 25). Another cognition test is the cognition performance test (CPT) evaluates cognition based on observation of the subject while they perform activities of daily living (ADLs) and instrumental activities of daily living (IADLs) activities (26). Dhillon et al. evaluated cognition by CPT tool in their study (19). The first attempt memory score (FAMS) is another test used for cognition and Alzheimer’s disease assessment which is used in an included study for cognitive function evaluation (7). Global cognition is a standardized score based on other assessments such as FAMS and Sala-Vila et al. reported their data using this score (21).

Most trials had a parallel design, and only one study was a cross-over RCT (6). Regarding the type of administered nuts, one study administered Brazilian nuts (20), two articles intervened with almonds (7, 19), and one administered walnuts (21).

Findings from the systematic review: Among the five RCTs assessing the scores of cognitions, two studies revealed a significant beneficial effect of nuts on cognition status (6, 20). The study by Rakic et al. suggests that higher amounts of nuts (3 oz./day compared to 1.5 oz./day) can be beneficial for cognition performance (7). However, two studies suggest that consuming nuts is not beneficial for cognition status (19, 21).

**Figure 2:**
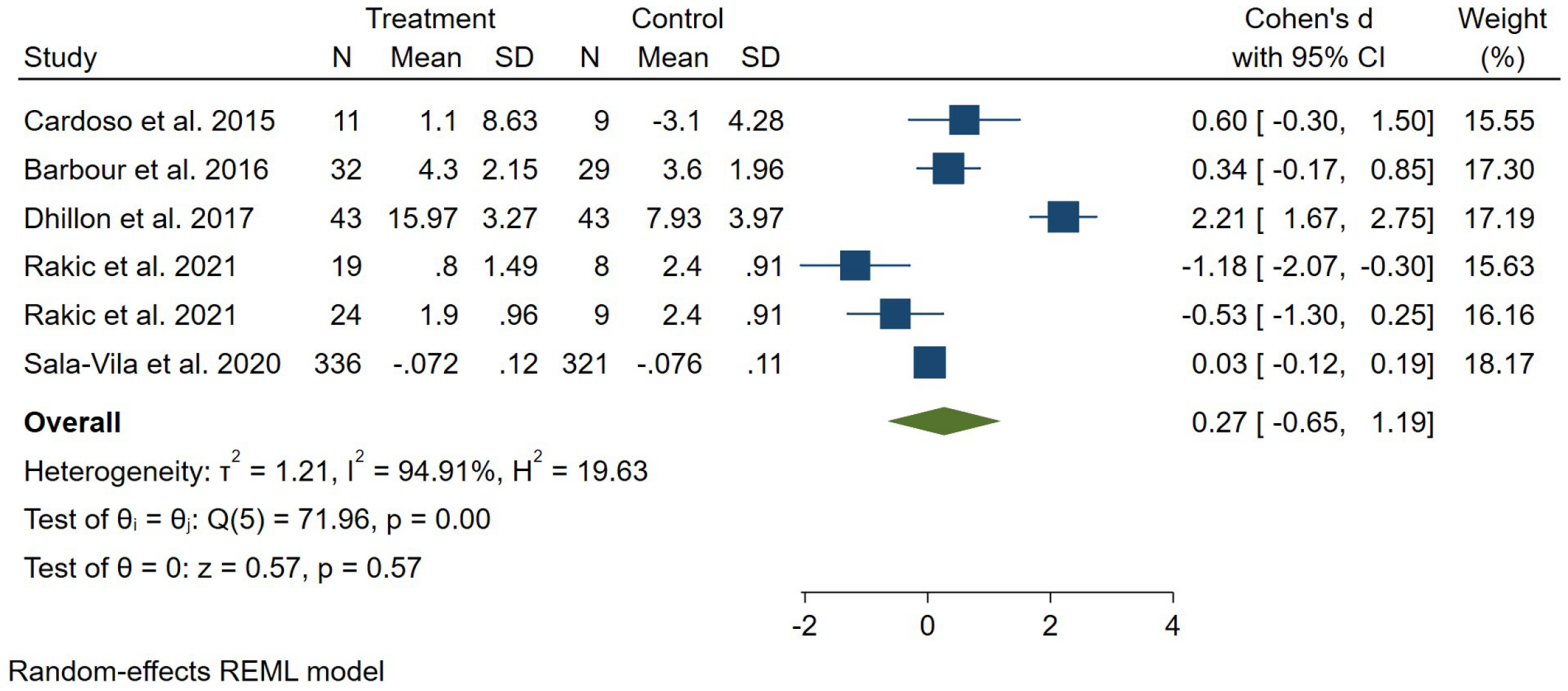
Forest plot for the effect of nuts consumption on cognition scores, expressed as standardized differences between intervention and control groups. Rakic et al. study had two intervention groups with different doses of almond, thus our analysis is based on six effect sizes of five trials (7). Horizontal lines represent 95% CIs. Diamonds represent pooled estimates from random-effects analysis. SMD, standardized mean difference; CI, confidence interval.

Findings from the meta-analysis nuts on cognition performance: In total, five RCTs with a total sample size of 928 subjects were included in the analysis (6, 7, 19–21). Combining six effect sizes from these RCTs states that nuts, compared with controls, resulted in an elevation in cognition scores [standardized mean difference (SMD): 0.27, 95% CI: −0.65 to 1.19, *p* = 0.57] (Figure 2).

However, our result was not significant (*p* = 0.57). In subgroup analysis, papers in which the duration of intervention was 6 months or less, showed a significant effect of nuts on cognition performance. Furthermore, there was evidence of a high between-study heterogeneity (*I*^2^ = 94.0, *p* < 0.001). We performed subgroup analyses, to detect potential sources of heterogeneity, (Table 2). Between-study heterogeneity was explained by the difference between the duration of studies based on our results from subgroup analyses. However, meta-regression did not support this result for the source of heterogeneity (*p* = 0.96). In addition to that, two of the included papers did not administer a placebo for their control groups thus, we performed a random effects analysis excluding those studies, and no significant change was observed in the final result [standardized mean difference (SMD): 0.17, 95% CI: −1.08 to 1.41, *p* = 0.793] (6, 22). Moreover, the study by Rakic et al. had 2 intervention arms with different dosas of almond and it was essential to separately include them in the analysis and divide the control between them. However, to ensure that including the two arms separately does not significantly affect our analysis, we conducted a random effect analysis after excluding the study resulting in no significant change [standardized mean difference (SMD): 0.79, 95% CI: −0.19 to 1.76, *p* = 0.114, *I*^2^ = 94] (7).

**Table 2:** Subgroup analyses for the effects of nut consumption on cognition performance.

	Effect size, *n*	SMD (95% CI)^a^	*p*-within^b^	*I*^2^ (%)^c^	*p*-heterogeneity^d^
Nut consumption on cognition performance					
Overall	6	0.27 (−0.65, 1.19)	0.57	94.0	<0.001
Study location					
Europe	3	−0.45 (−1.16, 0.26)	0.21	76	0.012
non-Europe	3	1.07 (−0.12, 2.25)	0.08	91	<0.001
Type of administered nuts					
non-Tree nut	1	0.34 (−0.17, 0.85)	0.19	0	0
Tree nut	5	0.25 (−0.89, 1.38)	0.67	95.4	<0.001
Duration of intervention					
≤6 months	4	0.24 (−1.21, 1.68)	0.75	95	<0.001
>6 months	2	0.13 (−0.29, 0.55)	0.53	31.32	0.23
Mean age					
<60	1	2.21 (1.65,2.75)	<0.001	0	0
>60	5	0.10 (−0.64, 0.43)	0.71	78.67	0.02

SMD, standardized mean difference; CI, confidence interval.

a Obtained from the random-effects model.

b Refers to the mean (95% CI).

c Inconsistency, percentage of variation across studies due to heterogeneity.

d Obtained from the *Q*-test.

**Figure 3:**
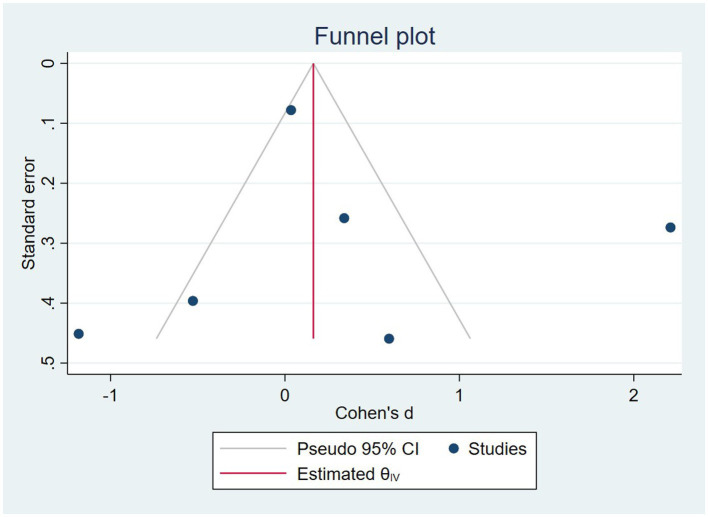
The funnel plot for publication bios of selected studies.

In the sensitivity analysis, excluding any single study did not affect the overall estimate of the effect of nuts on cognition (range of summary estimates: −0.86, 1.49). According to meta-regression, nuts can be more beneficial for younger adults compared to older ones and longer duration of study may increase the effect of them on cognition. However, this result was insignificant (Supplementary Table S4). In addition to that according to the funnel plot, no evidence of publication bias was observed (Figure 3). The same result was seen based on Egger’s test (*p* = 0.692).

## Discussion

In the current meta-analysis, we did not find a significant effect of nuts on cognition performance, however, we found an increasing effect of nuts on cognition performance. In subgroup analysis, one study which was conducted on adults with a mean age of 31 ± 13 had a significant effect on elevation cognition scores (18). All the other results in subgroup analyses were insignificant.

Nuts are classified as fruits consisting of two parts: a seed shell and a hard outer layer. They are known to be a calorie-dense food source and contain various types of nutrients. Generally, nuts are considered to have a good amount of fat, fiber, and protein. They are exceptionally beneficial for any diet as consuming them may help prevent certain diseases such as cancer and cardiovascular diseases. Nuts have a low glycemic index due to their high unsaturated fat and protein content, as well as low carbohydrate content. Unsaturated fats such as omega-3 fats can be beneficial for cognition by many pathways. They can reduce oxidative stress as a key factor for cognition decline. Additionally, they act as anti-inflammatory and pro-resolving mediators. Moreover, docosahexaenoic acid (DHA) as an omega-3 unsaturated fatty acid can prepare cells to counteract reactive oxygen species attacks by regulation of the nuclear factor erythroid 2 like 2 (NFE2L2) and heme-oxygenase-1 (HO-1) which is a downstream target protein for NFE2L2 (27). Fiber, another compound that is found in nuts, can be beneficial for cognition by short-chain fatty acids (SCFAs) conversion and regulation of the gut-brain axis and boost cognition (28). These are just a few possible mechanisms suggesting a beneficial effect on cognitive function following nut consumption. There are many more nutrients with multiple pathways supporting cognition. They have also been shown to be beneficial for cognitive function (29). A recent systematic review in 2020 found that consuming nuts can be beneficial for cognition, especially in individuals with cognitive impairment (8). Numerous observational studies are reporting an association between nut consumption and prevention of cognition decline a cross-sectional study by Arab & Ang showed that consuming a higher amount of nuts may significantly reduce simple reaction time test (walnuts with high certainty: mean difference: −17.4 ms, *p* = 0.031) (30). A prospective cohort with 5 years of follow-up on 2,613 middle-aged adults showed that higher consumption of nuts can boost cognitive function but not always significantly (31). There are other studies supporting these results (32, 33). However, some of them do not show a significant beneficial association between a higher amount of nuts and higher cognition scores (34–36). Similar to observational studies, interventional studies are controversial. Some clinical trials suggest a significant improvement in cognition performance with nut consumption. A 2016 study by Barbour et al. suggests that high-oleic peanut supplementation has the potential to regulate circulatory function in the brain and improve cognition in people with cognitive impairment (6). On the other hand, Mustra Rakic et al. found no significant difference in cognition improvement for consuming almonds among the three groups included in their study (7). However, the study included a large sample size of 928 participants. To reach a firm conclusion, studies on each type of nut should be conducted. As there were not many RCTs and each of them used a different type of nut, we were unable to perform a subgroup analysis on all different types of nuts in separate groups. The current meta-analysis did not have a significant result, but a significant result was found in subgroup analyses. Moreover, none of the included RCTs had reported baseline dietary consumption of nuts. Finally, heterogeneity between studies was high, but we could determine that the source of heterogeneity was the duration of the intervention by conducting subgroup analyses. However, meta-regression did not support the result from the subgroup analysis. Different types of nuts could be a source of heterogeneity but as it was mentioned earlier, we were unable to make a separate group for each type of nut. However, we conducted a subgroup analysis by comparing tree nuts and non-tree nuts.

In conclusion, in the current meta-analysis, we did not find a significant effect of consuming nuts on cognitive function scores. However, the analysis showed an elevation in scores following the consumption of nuts but not significant. It is suggested that future RCTs need to have a duration of 12 weeks minimum as it is unlikely that nuts can affect cognition for a shorter duration. It is clear that intervention longer than 12 weeks can be more accurate and is preferred. Moreover, as the number included studies were not many and they were heterogeneous, we were unable to perform a dose-response analysis and thus, we were unable to find an optimum dosage. Future trials and meta-analyses on them should be more focused on finding a preferable dosage for best efficacy. Finally, future studies should assess and report the baseline intake of nuts as it can be a very important confounder.

## Data Availability

The original contributions presented in the study are included in the article/Supplementary material, further inquiries can be directed to the corresponding author.
