# Supplementary material for: The effect of consuming nuts on cognitive function: a systematic review and meta-analysis of randomized clinical trials
Source: Front Nutr. 2024 Sep 4;11:1463801. doi: 10.3389/fnut.2024.1463801 (PMC11408291; doi:10.3389/fnut.2024.1463801)
Supplement: Supplementary file 2 [file Table_2.DOCX]

**Supplemental Table 2**: The terms used to search relevant publications on the effect of consuming nuts on cognitive function

| **In PubMed, Scopus, and ISI Web of Science** | | **n** |
| --- | --- | --- |
|  | 1. “nuts” |  |
|  | 2. “nut” |  |
|  | 3. “almond” |  |
|  | 4. “cashew” |  |
|  | 5. “tree nut” |  |
|  | 6. “peanut” |  |
|  | 7. “pecan” |  |
|  | 8. “pine nut” |  |
|  | 9. “pistachio” |  |
|  | 10. “macadamia” |  |
|  | 11. “peanut butter” |  |
|  | 12. “hazelnut” |  |
|  | 13. " walnut " |  |
|  | 14. (1 OR 2 OR 3 OR 4 OR 5 OR 6 OR 7 OR 8 OR 9 OR 10 OR 11 OR 12 OR 13) |  |
|  | 15. “ cognition “ |  |
|  | 16. “ executive “ |  |
|  | 17. “ executive function “ |  |
|  | 18. “cognitive control “ |  |
|  | 19. “intelligence” |  |
|  | 20. “memory” |  |
|  | 21. “attention” |  |
|  | 22. “metacognition” |  |
|  | 23. “cognitive” |  |
|  | 24. “cognition” |  |
|  | 25. “neuropsych”  26. “psychomotor”  27. “learning”  28. “language”  29. “executive function”  30. “attention”  31. “social cognition” |  |
|  | 32. (15 OR 16 OR 17 OR 18 OR 19 OR 20 OR 21 OR 22 OR 23 OR 24 OR 25 OR 26 OR 27 OR 28 OR 29 OR 30 OR 31) |  |
|  | (14 AND 32) **(With MeSH and Tiab)** **In PubMed** | 1610 |
|  | (14 AND 32) **In Scopus** | 1180 |
|  | (14 AND 32) **In ISI Web of Science** | 1670 |
|  | **Duplicate** |  |
| **In Google Scholar** | | 400 |
|  | Nut and cognition  By searching the above combination in this engine, we screened the first 400 relevancy ranked papers to avoid missing any eligible studies. |  |
| **Total** | | 3634 |
