# Supplementary material for: The effect of consuming nuts on cognitive function: a systematic review and meta-analysis of randomized clinical trials
Source: Front Nutr. 2024 Sep 4;11:1463801. doi: 10.3389/fnut.2024.1463801 (PMC11408291; doi:10.3389/fnut.2024.1463801)
Supplement: Supplementary file 3 [file Table_3.DOCX]

| Study | **Random sequence generation** | **Allocation concealment** | **Selective reporting** | **Blinding (participants and personnel)** | **Blinding (outcome assessment)** | **Incomplete outcome data** |
| --- | --- | --- | --- | --- | --- | --- |
| Cardoso et al. 2015 | **L** | **L** | **L** | **H** | **L** | **H** |
| Barbour et al. 2016 | **L** | **L** | **L** | **L** | **H** | **H** |
| Dhillon et al. 2017 | **L** | **L** | **L** | **U** | **U** | **L** |
| Rakic et al. 2021 | **L** | **L** | **L** | **H** | **H** | **H** |
| Sala-Vila et al. 2020 | **L** | **L** | **L** | **H** | **L** | **H** |

**Supplemental Table 3**: Results of risk of bias assessment for randomized clinical trials included in the current meta-analysis on the effects of nuts on cognition^1^

^1^We assessed each study for risk of bias. For this assessment, we used the Cochrane Risk of Bias Assessment tool (8). Domains of assessment included detection bias, allocation concealment, random sequence generation, performance bias, reporting bias, attrition bias, and other sources of bias. If a domain contained methodological flaws that may have affected the results, we scored it “high risk”, “low risk” if the flaw was deemed inconsequential, and “unclear risk” if there was not sufficient information to determine. If a study got “low risk” for all domains, we considered it as a high-quality study with totally low risk of bias.
