# Supplementary material for: The effect of consuming nuts on cognitive function: a systematic review and meta-analysis of randomized clinical trials
Source: Front Nutr. 2024 Sep 4;11:1463801. doi: 10.3389/fnut.2024.1463801 (PMC11408291; doi:10.3389/fnut.2024.1463801)
Supplement: Supplementary file 4 [file Table_4.DOCX]

| **Confounder** | **Effect size, n** | **Coefficient (95% CI)** | **P-value** |
| --- | --- | --- | --- |
| Mean age of participants | 6 | -0.05 (-0.12 to 0.02) | 0.16 |
| Duration of intervention | 6 | 0.5 (-2.13 to 2.23) | 0.96 |

Supplemental Table 4: Table for meta-regression test.
